# Medical Opinion and Movement

**Published:** 1908-06-27

**Authors:** 


### Medical Opinion and Movement.

OCCASIONALLY it happens that patients who are suspected to be phthisical have repeated attacks of coughing, but can produce no pulmonary sputum. It is always difficult to detect the tubercle bacilli in these cases, but Blume has devised a method for overcoming the difficulty in some of them. The patient is given a suitable piece of clean glass—a microscope slide, for example—with directions that he is to hold one side of it close to his mouth whenever he coughs. After two, three, or more days, as the case may be, the same slide having been used all the time, it is fixed and stained for tubercle bacilli in the ordinary way. Now and then the method is successful when the diagnosis would otherwise remain in doubt. To make doubly sure, a control slide may be kept in the patient's rooms and stained without having been coughed against; error from the presence of acid-fast bacilli in the atmosphere will thereby be avoided.

IN the *Bulletin Médicale* Guinard makes some observations upon the mode of action of certain drugs employed in the treatment of hæmoptysis in the tuberculous. He starts by laying it down as an axiom that intervention must take place as rapidly as possible, or it is useless; consequently only such drugs as modify the blood-flow are of use. Ergot and ergotin must be discarded as useless in such cases, because they produce no general effect on the circulation. Adrenalin, though a local vaso-constrictor, has, after absorption, no power whatever as a general hæmostatic, and similarly must be discarded. All drugs capable of developing a rapid and powerful vaso-dilatation, with consequent fall of blood-pressure, are useful in helping to stop a pulmonary hæmorrhage, and among this class of drugs amyl nitrite is one of the most important. Morphia is useful, given either with or after a vaso-dilator, not only because of its general soothing effect upon the patient, but also because it is a hæmostatic, relieves cough, and quietens respiration. Ipecacuanha in minute doses is excellent, especially after a hæmoptysis, and other drugs which will be found useful in such conditions, either as hæmostatics or adjuvants to these, are hydrastis, hamamelis, quinine, and digitalis. The writer also touches on forms of treatment other than by drugs, and is in favour of cupping and of ligature of limbs. The latter form of treatment tends to lower the general blood-pressure by causing blood to collect in the limb and so lessening the amount present in the general circulation.

DR. PAUL CARNOT discusses the medicinal treatment of gout in the light of recent chemical research. The principal indications recognised in the treatment of this malady are the restriction of uric-acid-producing food and the administration of drugs reputed capable either of dissolving uric acid deposited in the system or of destroying it or avoiding its formation. Laboratory and clinical experience are not altogether in harmony upon the solvent action of the various drugs. Alkalies, bicarbonate of soda, potash, and lithia have been shown *in vitro* to exercise a definite solvent effect upon uric acid, but *in vivo* their administration does not appear to augment the excretion of uric acid or to dissolve uric acid deposits. Their undoubted therapeutic efficacy must find some other explanation; possibly in some modification of the metabolic processes. Piperazine (diethylene-diamine) forms *in vitro* a highly soluble salt with uric acid, but *in vivo* the results of observers are very variable, but certainly do not indicate any increased uric acid elimination. Thyminic acid is of special physiological interest. The researches of Minkowski, Goto, and Kossel have shown that the nucleins decompose into uric and thyminic acids, and that thyminic acid is the physiological solvent of uric acid. Its presence keeps uric acid in solution in the blood, and it is suggested that the uric acid deposits in the gouty arise from a deficiency in thyminic acid. Thyminic acid is said to increase the excretion of uric acid in the urine, but nothing definite can be said at present in favour of its therapeutic efficacy for gout.

IT must be recognised that the physiological and chemical processes which determine the gouty manifestations cannot be represented by some simple chemical reaction in a test tube, and any principle of treatment, such as the administration of a solvent of uric acid, based upon such a reaction, must be inadequate. Dr. Carnot then turns to the recent researches upon the uricogenic and uricolytic ferments of the liver, and suggests that a further elucidation of their nature and action may lead to their use in a so-called hepatic opotherapy, by which one may be able to combat the gouty diathesis on more rational lines. According to these researches the decomposition of the nucleins to uric acid and urea takes place in stages by a series of ferments. These ferments are found in the various tissues of the body, but especially in the liver, and several have been distinguished. One of these, *nuclease*, liberates the amidopurines from the nucleic acid molecule. Others, such as desamidase, xanthin-oxidase, carry the oxidation process further, while the uricolytic ferment decomposes uric acid to glycocoll and urea. Much work has already been done by Horbaczewski, Spitzer, Wiener, and others in the elucidation of these complex chemical processes of the body, and it is work on these lines that may eventually lead to future advances in the therapeutics of gout.

THE *rôle* which is played by the micro-organisms found present in vaccine lymph has afforded subject for considerable discussion among bacteriologists. Whether they are to be regarded as a contamination pure and simple to be avoided or eliminated as far as possible, or whether they in some way contribute to the specific immunising activity of the vaccine, is the question at issue. More than one observer has found by careful examination and estimation of different strains of vaccine that the most active vaccines are also frequently those which contain the largest number and variety of microbes. M. B. L. Camus has endeavoured to throw light on the problem by determining whether the antivaccine serum, which destroys the active principle of the vaccine, acts also upon these adventitious microbes. He finds that the bactericidal power which belongs alike to normal serum and the serum of vaccinated animals is distinct from the antivaccine property of the serum of vaccinated animals, and that the bactericidal power of this latter serum can be destroyed by heating to 70° C., without affecting its antivaccine property. In this way a complete separation of the vaccine-virus from the adventitious germs can be effected. By mixing a vaccine solution with normal serum, the microbes disappear without destroying the vaccine. On the other hand, by adding to a vaccine solution the serum of a vaccinated animal, previously heated to 70° C., the vaccine is destroyed without affecting the microbes.

---

THE old and interesting question "What is Life?" with its consequent addendum "What is Death?" has interested philosophers more perhaps than it has done physicians—at least, its abtract discussion. The great "definitions" of Bichat, Spencer, and others are philosophical definitions, and hitherto few physiologists have ventured to approach with a new definition. Professor Nothnagel, the well-known Vienna pathologist, has lately attempted to define death by stating that it is "that phase in the being of an organism during which the manifestations of life by the body tissues either gradually lessen or are suddenly so strongly opposed that they cease entirely"—a statement which is as circumlocutory as a definition can be, and which brings nothing new to light. Nevertheless one should be grateful for its publication, since it has led another and much less well-known physician, Dr. Puppe, of Königsberg, to make it a text for a paper on death from the physician's point of view. This is in reality a study of the Death Agony, which the author considers as the gradual asphyxiation of the individual cells of the body, and his careful observations are interesting in so far as they throw light on some points which are as yet obscure.

---

ONE of these points is the question whether or not it is possible to prove that a person has died of suffocation—a matter of importance in many medico-legal cases. Leers has shown that interstitial pulmonary emphysema, formerly regarded as of value in proving that the individual has been forcibly deprived of air, is not a constant postmortem finding in cases where such forcible deprivation is known to have taken place before death, while it has been found in cases in which death was certainly not due to suffocation. Equally little reliable is the subserous ecchymosis, to which modern medico-jurisprudence denies the importance it formerly received, or the finding of fluid blood in the heart chambers. In fact, there is nothing characteristic, in the author's opinion, in the post-mortem findings after suffocation to enable the medical witness to state with absolute certainty that suffocation was the cause of death. Such findings may show a strong probability, which, combined with evidence of forcible suffocation, such as the presence of a string mark round the neck, may be accepted as conclusive, but alone they are worth little, as every one of them can be found in bodies in which "convulsive agony" has preceded death. The whole matter is condensed in the final clause in which the author lays down the principle that "in every case death is due to suffocation—*i.e.* asphyxiation—and the death agony is merely the sign of such commencing asphyxiation which may be due to primary heart failure, to cerebral anæmia, or to direct interference with the supply of air to the tissues."

---

THE medical treatment of exophthalmic goitre has not been very encouraging so far, and there is reason for the scepticism of some authorities who declare that no drug is of value in this affection, and that the only hope of success lies in early surgical interference. But so long as it is a fact that cases of exophthalmic goitre get well without surgical treatment, there is hope that the medical treatment of the disease may yet give encouraging results. While we know so little about the ætiology and pathology of the disease, there can be no dogmatic rules of treatment. All that can be done is to compare and contrast the results of specific methods. In doing this a certain amount of discrimination is necessary. A new method needs no defenders; its originators are only too keen to give their results to the world, and, quite unconsciously, such results are made to appear in a much better light than they often deserve. Failures are rarely recorded, although in new methods they teach far more, and are therefore far more valuable, than successes. In a full study of the treatment of exophthalmic goitre —a study that yet awaits some enthusiastic observer —such failures ought to be considered and weighed, and any paper that deals only with alleged brilliant successes should be looked upon with caution, tempered with a certain amount of distrust.

---

FOR that reason Tobias' recent article on the "Treatment of Basedow's Disease," founded on a series of cases treated at the author's sanatorium for physical treatment in Berlin, is of interest. It does not claim to be a record of uninterrupted success, and in some parts its reading is discouraging. The author, after a short introduction, in which he deals with the ætiology of the disease and incidentally with its pathology, dividing the cases into two main groups, the true exophthalmic and the false or pseudo-exophthalmic goitres, proceeds to consider the physico-dietetic treatment as carried out at the Institut für Physikalische Heilmethoden. In cases of pseudo-exophthalmic goitre the question of operation will have to be weighed. Tobias, in opposition to most physicians, is inclined to think that operation holds out "very good chances if it is undertaken early." In true exophthalmic goitre the question of operation is even more difficult to decide, and the author is less inclined to counsel surgical interference. He favours treatment with $x$-rays, a method which has not yet been thoroughly tried, but which appears to do good in some cases. Organotherapy has not been very successful, and the purely medicinal treatment the author does not approve of. He attaches his approval mainly to the physico-dietetic treatment, and in the course of an extended article fully considers its advantages and demerits.

---

In the first place the patients ought to be carefully nourished, and "in general the rules that should be conformed to in the treatment of neurasthenic patients hold good in the treatment of cases of Basedow's disease." A diet fairly rich in fats and carbohydrates should be ordered; a purely vegetable diet often does more harm than good. On the contrary, a vegetable-milk diet with periodical—three to six times weekly—variations of fish or meat, is to be recommended. Forcible feeding may be necessary in a few cases, and the author forbids alcohol entirely. The main features in the diet are a sufficient amount of fat and carbohydrate and a pleasant variety. The author recommends a moderately high, equable climate, sea air and fresh air in quantity. He has found benefit result from hydro-therapeutic treatment, the baths being usually cold, or cold compresses over the gland being used. In severe cases great caution must be employed in giving these cold baths or even the cold-water compresses, and it is better to begin treatment with tepid or even moderately hot baths, gradually reducing the temperature. In addition to this water treatment, massage is recommended, but this must be carried out in a proper and carefully regulated manner, in accordance with systematic rules, and strictly supervised by the medical attendant. Gymnastic exercises do not seem to have much effect upon the course of the malady, but the author has found benefit result from deep respiratory exercises, which in every case must be conducted in the open air or at least before an open window. Finally, electric treatment, either directly or by means of faradic baths, is of use. In conclusion, the author remarks that the serum treatment has still to be proved of value; operative treatment is of value, more especially in such cases where the local condition, the goitre, dominates, while the physico-dietetic method appears to be worthy of consideration in every case, even though it does not always prove successful.

---

The association of scarlet fever with measles in children affords the subject of some recent papers by M. P. Lereboullet and M. Orsoni. The influence which the one disease exercises upon the other varies according to the time relations of the incidence of the two diseases. If the two diseases are contracted at the same time, the incubation period for scarlet fever being shorter leads to the appearance of the symptoms of this disease before the onset of measles, and the authors emphasise the fact that it is precisely this type of association of the two fevers which offers the most serious prognosis. Naturally the greater the interval between the appearance of the two diseases the less is the course of the one influenced by the other. When scarlatina follows measles, the prognosis is not serious, although a post-morbillic broncho-pneumonia may be aggravated thereby. When the two fevers appear simultaneously, the mixed eruption may present special characters affording difficulties in diagnosis. Complications and sequelæ may arise, due to the one affection or the other; but the fact of the appearance of the two diseases together does not seem to seriously affect the course or prognosis of the case. In the cases referred to above, when scarlatina precedes measles by a few days' interval, broncho-pneumonia in a severe form is very likely to follow. In explanation of this fact, it is suggested that the infective condition of the scarlatina throat is responsible. From the onset of the disease virulent streptococci are found in the throat and are probably responsible at least for the greater part of the complications which arise, if not for the disease itself. With such a focus of infection close at hand the ordinary bronchial catarrh of measles may easily and rapidly develop, as is found to be the case, into a diffuse and fatal broncho-pneumonia. The therapeutic indication is, of course, constant and thorough disinfection of the throat.

---

Hæmatemesis in cases of typhoid fever has not attracted so much attention as the more common complications, and Professor Schlesinger's recent contribution to the literature on the subject is therefore welcome. In most text-books no mention whatever is made of hæmatemesis as a complication of enteric, and the practitioner who comes across it in private practice may very well confess his complete ignorance of its value as a prognostic sign. It is not by any means a frequent complication, although at autopsies gastric ecchymoses and sub-mucous hæmorrhages near the pylorus are not rare. In Schlesinger's two cases, observed in the medical department of the Royal Franz Joseph Hospital in Vienna, the hæmatemesis ushered in the disease in one case. The patient had aortic regurgitation, and had already been in hospital, three years previously, for gastric symptoms. A well-marked Widal reaction was present, with leucopenia, and the usual signs of enteric fever. Frequently repeated tests for traces of blood in the fæces were negative. The second case was that of an eighteen-year-old girl, who during the second week of her illness was seized with sudden vomiting and hæmatemesis, accompanied by collapse. Adrenalin and camphor injections were given, but the patient never rallied, and died sixteen hours after the onset of the vomiting. At the autopsy severe enteric lesions were made out, but the intestine itself was free from blood, while the stomach was almost full of it, and there were numerous sub-mucous hæmorrhages throughout the extent of the latter organ. The author concludes that such cases fall into two well-marked groups—cases of initial and cases of terminal gastric hæmorrhage, of which the second class is much more important from a prognostic point of view.

